# Personality Effects on Romantic Relationship Quality through Friendship Quality: A Ten-Year Longitudinal Study in Youths

**DOI:** 10.1371/journal.pone.0102078

**Published:** 2014-09-18

**Authors:** Rongqin Yu, Susan Branje, Loes Keijsers, Wim H. J. Meeus

**Affiliations:** 1 Research Centre Adolescent Development, Utrecht University, Utrecht, the Netherlands; 2 Department of Developmental Psychology, Tilburg University, Tilburg, the Netherlands; University of Granada, Spain

## Abstract

This study examined whether individuals with different personality types (i.e., overcontrollers, undercontrollers, resilients) had different friendship quality development throughout adolescence. It also investigated whether personality types were indirectly related to romantic relationship quality in young adulthood, via friendship quality development in adolescence. The study employed six waves of longitudinal questionnaire data from Dutch youths who had a romantic relationship when they were young adults. Two age cohorts were followed, from 12 to 21 years and from 16 to 25 years, respectively. Findings showed that resilients reported higher mean levels of friendship quality during adolescence (i.e., more support from, less negative interaction with and less dominance from their best friend) than both overcontrollers and undercontrollers. Through the mean levels of friendship quality throughout adolescence, resilients indirectly experienced higher romantic relationship quality during young adulthood than both overcontrollers and undercontrollers. Thus, results provide support for a developmental model in which adolescent friendship quality is a mechanism linking personality types with young adulthood romantic relationship quality.

## Introduction

Friendships and romantic relationships are both important for psychosocial development in adolescence and young adulthood [1], [2]. Both of these social relationships are voluntary and reciprocal, and thus have important characteristics in common. Friendships and romantic relationships also vary in the developmental significance over the life course, however. Whereas friendships are often the first voluntary and reciprocal relationship in a persons’ life, and fulfill important developmental needs during childhood and adolescence, romantic relationships typically become more salient during emerging adulthood [3], [4]. As such, friendships may serve as a learning ground for later romantic relationships [5], [6]. In other words, success in friendships is likely to affect the mastery of romantic relationships.

Not all adolescents and young adults develop optimal and satisfactory relationships. For instance, it has been proposed and empirically demonstrated that personality affects both individuals’ friendships and romantic relationships [3], [7], [8]. Generally, adolescents with a resilient personality tend to have both better friendships in adolescence and better romantic relationships in young adulthood [9], [10]. However, there are some gaps in our understanding of how these differences arise over the course of adolescence and emerging adulthood. Firstly, although prior research has shown linkages between personality and friendship quality, findings are inconclusive, mainly due to the fact that linkages have been studied across limited developmental periods. Secondly, to our best knowledge, it is unknown whether personality affects later romantic relationship quality through earlier friendship quality development, despite the fact that developmental “spill-over” between friendships and romantic relationships is plausible. The present study attempted to fill these gaps by drawing on insights from individual personality differences and developmental perspectives together.

### Personality Types and Quality of Social Relationships

Both friendship and romantic relationship quality might vary as a function of personality. People with different personality traits can differ in their motivations, as well as their interactions in and perceptions of social relationships [11], [12]. For instance, agreeable persons tend to have stronger motives for maintaining positive social relationships and try to minimize interpersonal disputes by being less aggressive, and therefore experience higher relationship quality [13], [14]. Additionally, people who are low in emotional stability are more likely to interpret ambiguous relationship scenarios in a more negative way, and to experience lower relationship quality [15]. Hence, there are clear empirical indications that personality is indeed linked to the quality of social relationships. However, variable-centered studies can only partially address this issue. Such an approach cannot unravel differences in social relationship quality for people who are both agreeable and emotionally unstable, for instance. Since separate dimensions of personality do not describe the person as a whole, there is a growing recognition of the need for a person-centered approach to understand personality and its associations with individuals’ relational outcomes [16], [17].

#### Personality types

One of the most often applied person-centered approaches to personality was based on Block and Block’ (1980) theory on ego-control and ego-resilience. Ego-control refers to the tendency to contain versus express motivational impulses, and ego-resiliency refers to the tendency to respond flexibly to environmental demands. Studies have suggested that three personality types–resilients, undercontrollers, and overcontrollers–could be constructed as specific combinations of ego-control and ego-resilience [13], [18]. Specifically, resilients are characterized by a high level of ego-resiliency and a medium level of ego-control. Overcontrollers and undercontrollers both have a low level of ego-resiliency, but differ on ego-control. Overcontrollers have a high level of ego-control and undercontrollers have a low level of ego-control [17], [18]. Several studies have revealed that these three personality types can be reliably constructed using Big Five personality traits in adolescents [19], [20]. Resilients generally have higher scores on all five dimensions: Extraversion, agreeableness, conscientiousness, emotional stability, and openness. Undercontrollers are characterized by lower conscientiousness and agreeableness, compared to others. Overcontrollers typically have relatively lower extraversion and lower emotional stability, compared to others, yet comparable agreeableness as Resilients [17], [18], [20]. We will adopt this personality classification to understand how individuals with these three distinct personality types vary in their social relationships.

#### Relationship quality

Social relationships have both positive and negative features [21], [22]. On the one hand, social relations can be salient sources of support by providing companionship, intimacy, assistance, and guidance. On the other hand, relationships provide a context for negative interactions, such as conflict and antagonism among interpersonal partners. A third feature that needs to be distinguished to understand relationships is the perceived dominance in the relationship, that is, the extent to which one is controlled and dominated by the other [23]. Although there are other aspects of a social relationship that are important, we focus on these three dimensions as they together encompass both positive and negative features of a social relationship. Moreover, these three dimensions provided a common conceptual framework among various types of relationships in the social network [24], [25]. Therefore, in the current study, perceived support from, negative interaction with, and perceived dominance from interpersonal partners, are the key dimensions adopted to typify friendships over the course of adolescence and romantic relationships in emerging adulthood.

### Personality Types and Friendship Quality in Adolescence

Research has shown that individuals with different personality types might have distinct patterns of friendship quality. Resilients tend to have better quality of friendships than both overcontrollers and undercontrollers [7], [10]. More specifically, cross-sectional studies using adolescent samples with average ages varying from 12 to 17 years have shown that resilients perceived more support from their friends than both overcontrollers and undercontrollers, whereas between the latter two there were no significant differences [26], [27]. A longitudinal study examining this link among adolescents from 13 to 16 years showed similar findings [10]. Furthermore, one study followed adolescents from 12 to 16 years and showed that overcontrollers and undercontrollers were equally high in conflict frequency and hostile conflict management, and they were both significantly higher in these two aspects than resilients [28]. Former research also provides some evidence regarding different levels of perceived dominance from friends for youths with different personality types. Overcontrollers experienced more coercion from their friend, and they were more likely to comply with their best friend in conflict and be influenced by their best friend’s delinquency than resilients [10], [28], [29]. Moreover, overcontrollers scored significantly lower than resilients on social potency which describes the propensity to enjoy leadership roles and desire to influence others [9]. For undercontrollers, results are less consistent: Similar to overcontrollers, they experienced more coercion from their friend and were more likely to comply with their best friend during conflict than resilients [10], [28]. They did not differ from resilients in their tendency to influence their friend with their delinquent behavior, however, nor in their level of social potency [9], [29]. In sum, both overcontrollers and undercontrollers seem to perceive less support and more negative interaction in friendships than resilients, and overcontrollers tend to perceive more dominance from friends than resilients. Results are mixed as to whether undercontrollers differ from resilients regarding perceived dominance from friends. All of these studies were limited to early to middle adolescents, however, and predominantly studied differences in terms of mean levels. The current study will examine personality differences in the mean levels of, and the developmental changes in, friendship quality among adolescents from 12 to 20 years.

### Personality Types and Romantic Relationship Quality in Emerging Adulthood

Individuals with different personality types also differ in romantic relationship quality. Personality types identified in early childhood were found to predict the quality of romantic relationships in young adulthood [9], [30], [31]: Undercontrollers, compared to resilients, reported lower quality of romantic relationships, as indicated by lower emotional support and warmth (e.g., intimacy and trust, acceptance, and exchange of personal thoughts and feelings), higher levels of conflict, and a more unequal balance of power in the relationship. Quite surprisingly, overcontrollers had similarly high romantic relationship quality as resilients in these three studies, despite the fact that overcontrollers generally reported lower friendship quality. Perhaps this absence of differences between overcontrollers and resilients can be explained by the fact that previous studies have assessed personality at one point in early childhood, rather than accounting for adolescent personality and its maturation over the course of adolescence [17], [20]. As personality develops during childhood and adolescence, personality measured during adolescence might be differently related to romantic relationship quality in young adulthood. Using a developmental personality typology to capture the normative changes of personality in adolescence might help in drawing a more comprehensive picture about the link between personality types and romantic relationship quality.

### Adolescent Friendships and Romantic Relationships in Emerging Adulthood

Friendships offer an important training ground for developing capacities and expectations for later romantic relationships [6], [32], [33]. Consistent with these theoretical ideas, several longitudinal studies have shown positive linkages between adolescent friendships and emerging adult romantic relationships [3], [34]–[36]. These studies, with time intervals ranging from 4 years to 7 years, revealed significant associations of weak to moderate effect size for various indicators of quality of friendships and romantic relationships, such as social support, commitment, and hostility. Specifically, individuals’ support from friends at age 15 and 17 was positively related to support from romantic partners at age 21 [36], and more support from friends at 16 years was predictive of longer-term committed romantic relationship from 18 to 25 years [35]. Similarly, relational commitment in adolescent friendships was predictive of relational commitment to their romantic partner in emerging adulthood [3]. Moreover, positive conflict resolution with friends at age 16 was related to more commitment and less hostility in young adults’ romantic relationships [34]. In sum, existing studies have consistently provided support for the idea that friendship experiences during adolescence might contribute in important ways to the quality of romantic relationships in emerging adulthood.

### Personality Types, Adolescent Friendships, and Romantic Relationships in Emerging Adulthood

No prior research has examined why adolescents with different personality types might vary in their quality of romantic relationships in emerging adulthood. As introduced above, previous studies have provided some evidence about the linkages between personality types and friendship quality, although they predominately focused on early to middle adolescents. Additionally, prior research has shown significant linkages between quality of adolescent friendship and young adults’ romantic relationships. These linkages suggest a natural progression for romantic relationship development, in which youths practice principles of volition and reciprocity in friendships and generalize related abilities and expectations to later romantic relationships. This developmental trajectory forms the rationale for an indirect effect of personality types on later romantic relationship quality, through earlier friendship quality. More specifically, we proposed that there would be an indirect pathway, such that adolescent personality types were associated with differential development of friendship quality during adolescence, which in turn would be associated with romantic relationship quality.

### The Current Study

Overall, this study aimed to test whether adolescent personality types were related to differential mean levels and developmental changes in friendship quality throughout adolescence (aim 1) and whether, through these differences in adolescents’ friendship quality, adolescent personality types would indirectly predict romantic relationship quality during young adulthood (aim 2).

## Method

### Participants

Participants were 524 Dutch youths who had a romantic relationship during young adulthood. They were part of an ongoing longitudinal study CONAMORE (CONflict And Management Of RElationships study), which in total consists of 1313 participants divided into two age cohorts. We collected data for one cohort from age 12 onwards (i.e., younger cohort; *n* = 923), and for the other cohort from age 16 onwards (i.e., older cohort; *n* = 390), respectively. For the current study, we used data from the annual measurements Wave 1 to Wave 5, collected from 2001 to 2005, and Wave 6 data, collected in 2010. Thus, participants were followed for ten years, from 12 to 21 years for the younger cohort and from 16 to 25 years for the older cohort. Because the aim of the study was to explain the quality of romantic relationships in early adulthood, only participants who had a romantic relationship during the sixth measurement wave (Wave 6) were included (*n* = 524). That is, 343 participants (227 girls) out of the initial 923 participants from the younger cohort, and 181 participants (112 girls) out of the initial 390 participants from the older cohort were included. The mean ages of these subsamples at Wave 1 were 12.37 years (*SD* = 0.56) for the younger cohort and 16.56 years (*SD* = 0.81) for the older cohort. For both cohorts, the ethnic compositions were 91.9% Dutch and 8.1% ethnic minorities. Regarding education levels at Wave 6, 266 participants (77.6%) from the younger cohort and 84 participants (46.4%) from the older cohort were completing further education. There were significant differences between participants who had a relationship at Wave 6 and those who did not, but all of these differences were of small effect size. Specifically, the percentages of girls and native Dutch in the group with a romantic relationship were significantly higher than those in the group without a relationship at Wave 6 (χ^2^ [*N = *1313, 1] = 60.92, *p*<.001, φ = .22; χ^2^ [*N = *1267, 1] = 25.41, *p*<.001, φ = .14). Moreover, after controlling for gender differences, young adults with a romantic relationship at Wave 6 perceived more support from their best friend (F [1, 1099] = 10.61, *p*<.001, *r* = .10), less negative interaction with their best friend, and less dominance from their best friend (F [1, 1105] = 7.31, *p* = .01, *r* = .08 and F [1, 1083] = 4.83, *p = *.03, *r* = .07, respectively), than young adults without a romantic relationship. There was a significant difference in the distributions of personality types among those who had a relationship at Wave 6 and those who did not (χ^2^ [*N = *1313, 2] = 9.10, *p* = .01, φ = .08). Undercontrollers were significantly less likely to have a romantic relation at Wave 6 (χ2 [N = 1313, 1] = 4.35, *p* = .04, φ = .06), whereas resilients were significantly more likely to have a romantic relation at Wave 6 (χ2 [N = 1313, 1] = 8.46, *p*<.001, φ = .08).

### Procedure

Participants were initially included from a number of randomly selected high schools in the province of Utrecht, The Netherlands. Participants and their parents received an invitation letter, describing the research project and goals, and giving the option of not participating in the study. More than 99% of the approached adolescents decided to participate in our study. From Wave 1 to Wave 5, our participants annually filled in various questionnaires at school after school hours. Participants who changed schools during measurement of Waves 1 to 5 and participants at Wave 6 filled in the questionnaires at their homes. Trained assistants gave verbal instructions to participants in addition to written instructions in the questionnaires. Confidentiality of participants’ given answers was assured explicitly before participation. Participants received €10 as a reward for their participation from Wave 1 to Wave 5, and €30 in Wave 6.

For participation in the present study, written informed consent was obtained from adolescents and their parents, and also from all the participating schools. Treatment of participants was in accordance with the ethical standards of the APA and this study was reviewed and approved by the ethical-medical committee of University Medical Centre Utrecht, the Netherlands.

### Measures

#### Adolescents’ personality types

Adolescents’ personality was assessed annually for five years with the Quick Big Five questionnaire [37], [38]. Thirty personality makers were used to assess five personality dimensions (each with 6 items): Extraversion (e.g., “talkative”), Agreeableness (e.g., “sympathetic”), Conscientiousness (e.g., “systematic”), Emotional stability (e.g., “worried”, reverse-scored), and Openness to experience (e.g., “creative”). Adolescents rated their personality on a 7-point Likert scale ranging from 1 (*very untrue*) to 7 (*very true*). Prior research have reported adequate reliability and validity of this scale [39]. In the current study, across Wave 1 to Wave 5, Cronbach’s alphas ranged from .80 to .87 for Extraversion, from .81 to .87 for Agreeableness, from .85 to .91 for Conscientiousness, from .80 to .83 for Emotional stability, and from .76 to .77 for Openness to experience. Several studies have shown that Block and Block’s (1980) three personality types (i.e., overcontrollers, undercontrollers, and resilients) can be constructed directly from the Big Five dimensions [17], [18], [20]. An earlier study constructed personality types with Latent Class Growth Analysis (LCGA; [40]) on the original 1313 cases, including the current sample [41]. The LCGA indicated that a three-class solution fit the data the best and the entropy was .91, which indicated a high accuracy of classification [42]. Therefore, in the current research, we adopted that study’s classification of personality types (See [41] for specific scores on Big Five traits for each personality type). In our sample, there were 120 overcontrollers, 78 undercontrollers, and 145 resilients among the 343 participants in the younger cohort. There were 57 overcontrollers, 53 undercontrollers, and 71 resilients among the 181 participants in the older cohort.

#### Friendship and romantic relationship quality

Participants’ friendship quality from 12 to 20 years (i.e., from Wave1 to Wave 5) and romantic relationship quality during young adulthood (i.e., 21 and 25 years at Wave 6) were assessed with Network of Relationships Inventory (NRI; [43]). This inventory measures participants’ perceptions of support from their best friend or romantic partner, negative interaction with their best friend or romantic partner, and perceived dominance from their best friend or romantic partner. Support was assessed with twelve items, including items from different subscales tapping into companionship, instrumental aid, intimacy, nurturance, affection, admiration, and reliable alliance in friendship or in romantic relationship. A sample item was “How often do you turn to this person for support with personal problems?” Negative interaction was measured with six items from two subscales tapping conflict and antagonism. A sample item was “How much do you and this person get upset with or mad at each other?” Perceived dominance was assessed with six items from two subscales tapping the extent to which adolescents were controlled and dominated by their best friend or romantic partner. A sample item was “How often does this person get his/her way when you two do not agree about what to do?” Participants reported their friendship and romantic relationship quality on a 5-point Likert scale, ranging from 1 (*never*) to 5 (*always*). The NRI has good predictive, factorial, and construct validity (Furman, 1996). In the current study, across the five waves, Cronbach’s alphas ranged from .91 to .93 for perceived support from best friend, from .81 to .87 for negative interaction with best friend, and from .81 to .86 for perceived dominance from best friend. At Wave 6, Cronbach’s alphas were .92 for perceived support from romantic partner,. 90 for negative interaction with romantic partner, and .88 for perceived dominance from romantic partner.

### Statistical Analyses

**Figure 1:**
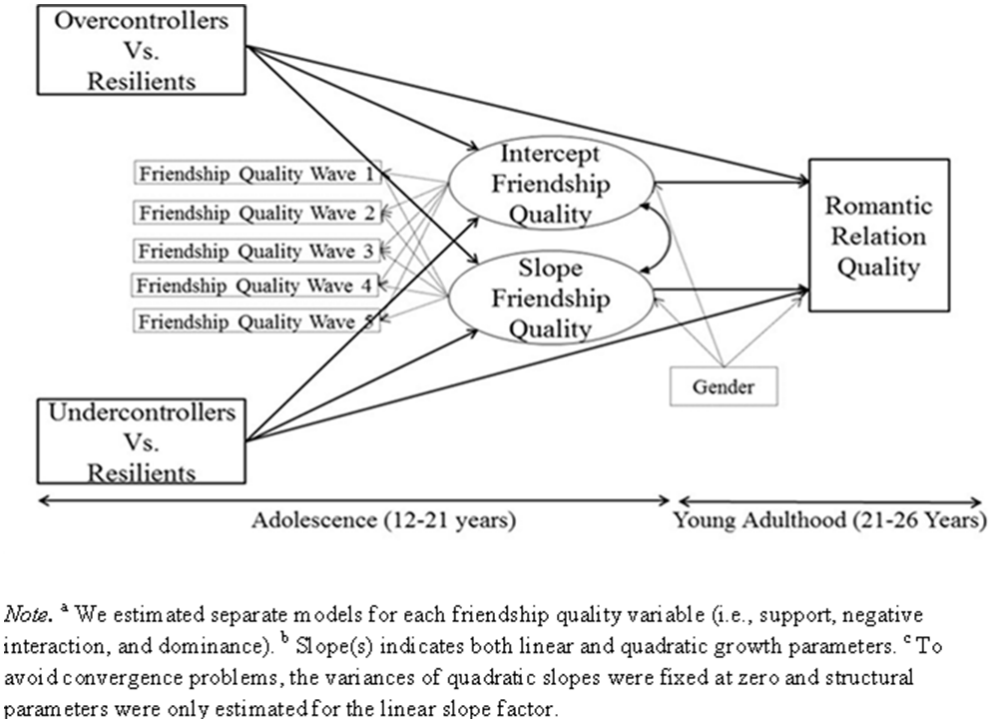
Structural Equation Model Testing the Relations between Adolescent Personality Types, Adolescent Friendship Quality Development, and Young Adulthood Romantic Relationship Quality.

All research questions were tested within comprehensive models existing of three groups of variables: 1) adolescent personality types (determined by fives waves of personality data); 2) latent growth factors (i.e., intercepts and slopes) capturing development of adolescent friendship quality across five waves; and 3) emerging adults’ romantic relationship quality. We estimated separate models for each friendship and romantic relationship quality variable. Age cohort was used as a grouping variable. In the model, depicted in Figure 1, adolescent personality types were entered as two dummy variables (i.e., overcontrollers vs. resilients and undercontrollers vs. resilients, with resilient personality type as a reference category coded as 0). We explored models including a dummy variable for the comparison between overcontrollers and undercontrollers. As only one out of twelve comparisons was significant (in the younger age cohort, overcontrollers experienced higher dominance from friends than undercontrollers), we did not further include results of these models. To test for the proposed structural linkages among these variables, we added paths from the personality dummies to the latent growth factors of friendship quality, paths from adolescent personality to romantic relationship quality in emerging adulthood, and paths from the latent growth factors of adolescent friendship quality to emerging adults’ romantic relationship quality. We additionally controlled for gender on the intercepts and slopes of adolescent friendship quality and on young adulthood romantic relationship quality. The models were tested in M*plus* [44] using a maximum likelihood (ML) estimator.

We first determined the shape of growth in friendship quality during adolescence. To that end, we compared the chi-square values of models including a linear and quadratic growth to capture changes in friendship support, negative interaction, and dominance [45]. The tests for negative interaction and dominance indicated that adding quadratic slopes significantly improved model fit (i.e., a significantly lower chi-square value; Δχ^2^ [N = 524, 2] = 25.35, *p*<.001 and Δχ^2^ [N = 524, 2] = 18.61, *p*<.001, respectively). For perceived support, the model with quadratic slope had a similar fit as the linear model (Δχ^2^ [N = 524, 2] = 3.21, *p* = .20). However, to facilitate the comparability between models across three friendship quality indicators, we chose models with both linear and quadratic slopes. To avoid convergence problems, the variances of quadratic slopes were fixed at zero.

After determining the shape of the growth of friendship quality, we tested whether parameters in the models could be constrained to be equal across cohorts, again by using chi-square difference tests [45]. These parameters included means, variances, and covariances of intercepts and slopes of friendship quality, and all structural regression paths in the models. Because the variance of the quadratic slope was constrainted at zero, no structural parameters could be estimated with this growth factor. In the final models, we constrained each parameter to be equal across two age cohorts that did not result in a significant decrease in chi-square value compared to the unconstrained model. All of the difference tests can be obtained from the first author upon request.

In addition, to evaluate the indirect effects of adolescent personality types on young adulthood romantic relationship quality through initial levels and developmental changes of friendship quality, the bias corrected bootstrapping method proposed by Preacher and Hayes [46] was used, using 10000 bootstrap resamples.

To evaluate the overall goodness of fit of the model, we used the Comparative Fit Index (CFI), the Tucker-Lewis Index (TLI), the Root Mean Squared Error of Approximation (RMSEA), and the Standard Root Mean Square Residual (SRMR). CFI and TLI values of .90 and above, and RMSEA and SRMR values of less than .08 are considered to indicate acceptable fit [47], [48].

## Results

**Figure 2:**
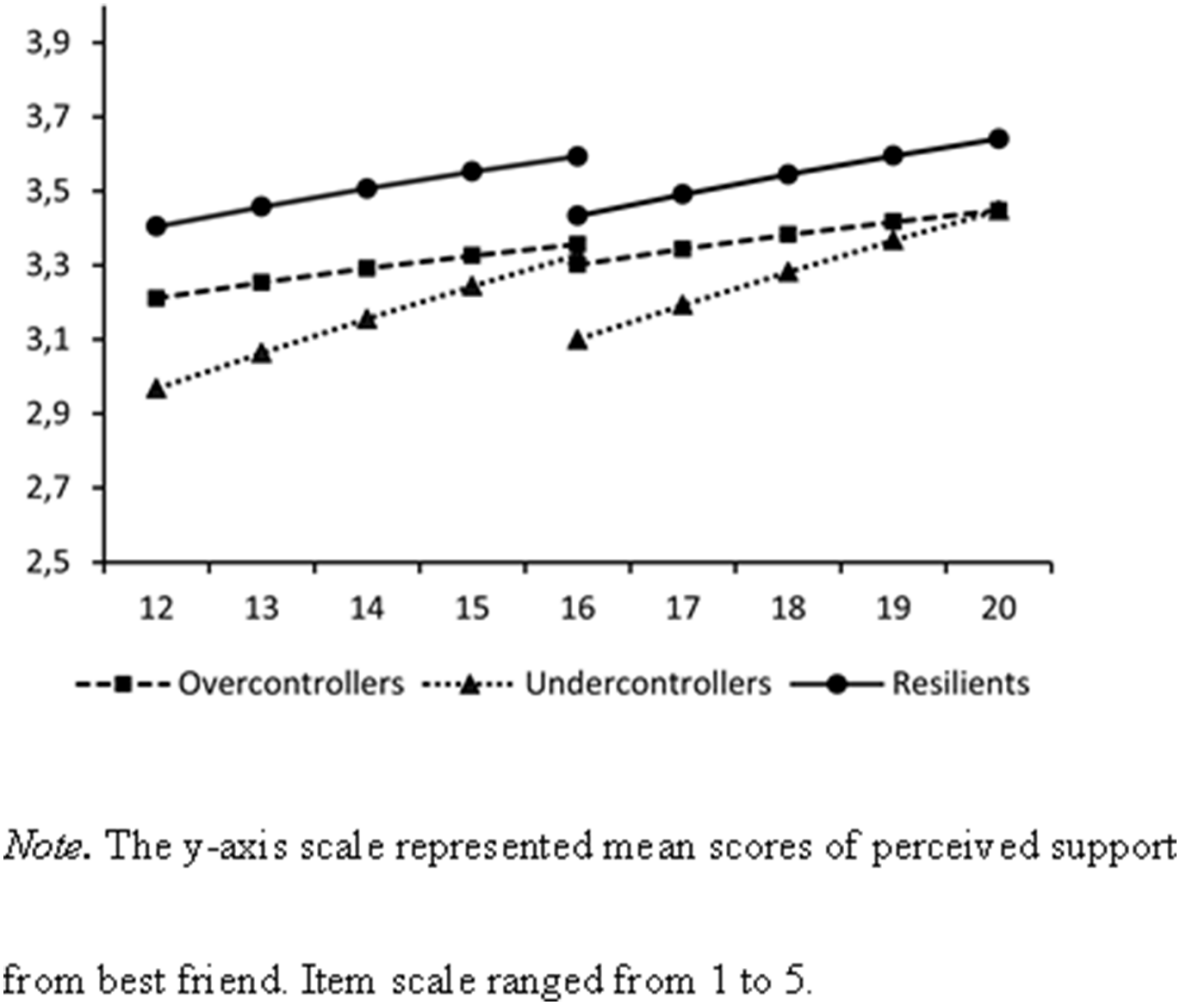
Estimated Developmental Changes in Adolescent Friendship Support by Adolescent Personality Types.

**Figure 3:**
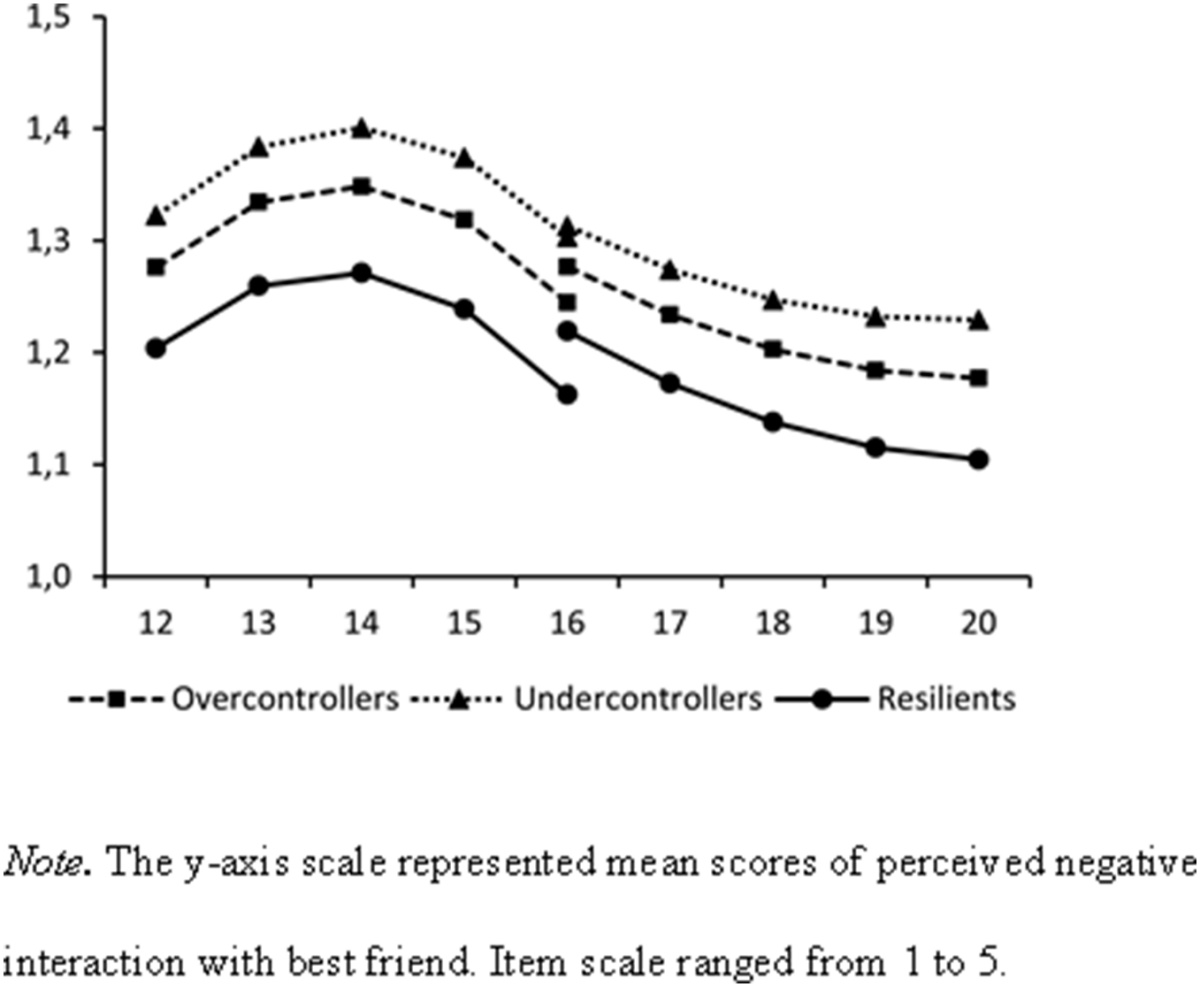
Estimated Developmental Changes in Adolescent Friendship Negative Interaction by Adolescent Personality Types.

**Figure 4:**
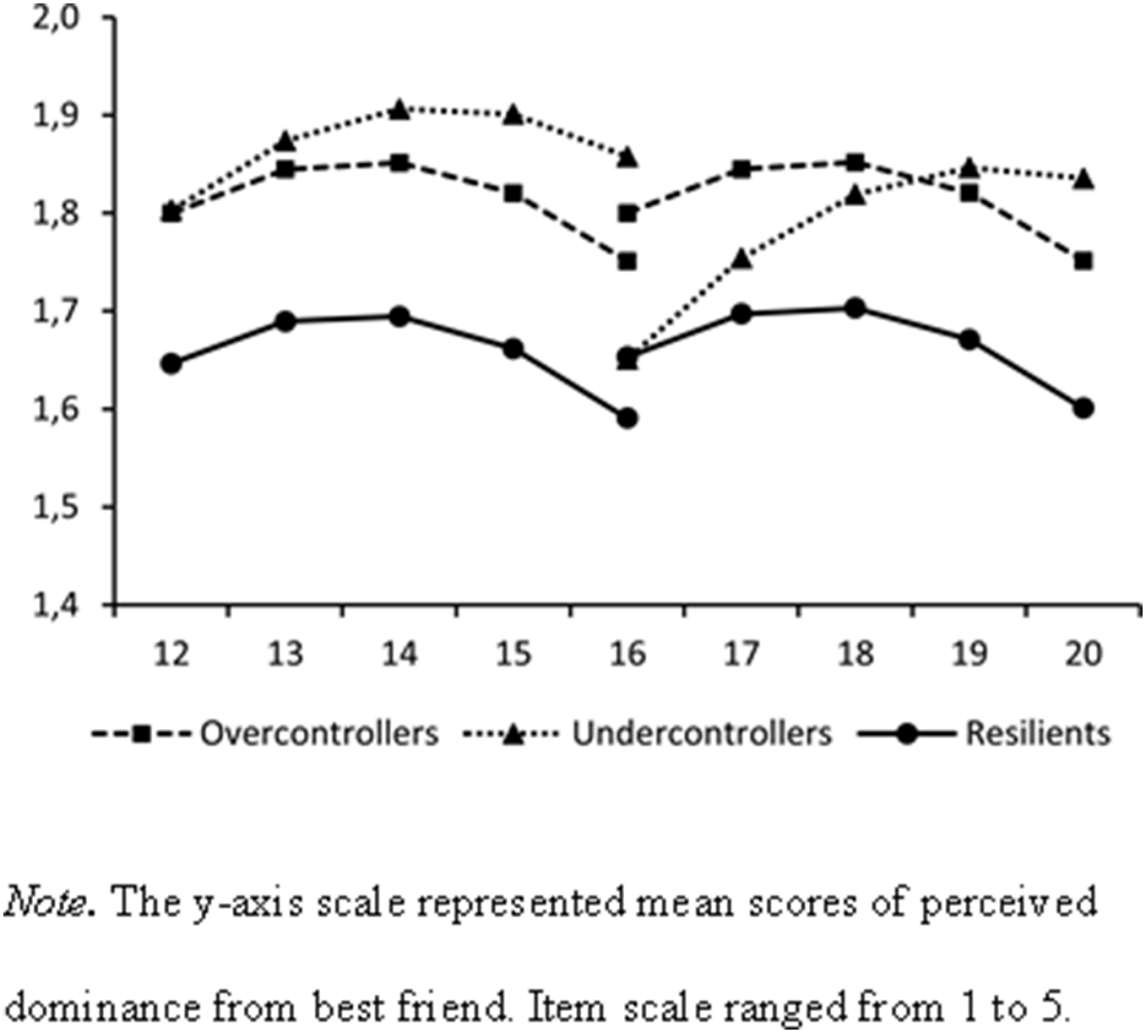
Estimated Developmental Changes in Adolescents’ Perceived Dominance from Best Friend by Adolescent Personality Types.

Table 1 presents the means and standard deviations of Wave 1 to Wave 5 adolescent friendship quality and Wave 6 young adulthood romantic relationship quality, for each adolescent personality type (i.e., overcontrollers, undercontrollers, and resilients). Table 2 presents bivariate intercorrelations between relationship quality indicators. Table 3 and Figures 2–4 present the results of our final structural equation models. These models all had an acceptable model fit, with CFIs and TLIs higher than .90, and RMSEAs and SRMRs lower than .08.

**Table 1:** Means and Standard Deviations of the Observed Values of Adolescent Friendship Quality and Young Adulthood Romantic Relation Quality by Adolescent Personality Types.

Relation QualityIndicator	Early to Middle Adolescent Friendship Quality					RomanticRelationQuality	Middle to Late Adolescent Friendship Quality					RomanticRelationQuality
	12 years	13 years	14 years	15 years	16 years	21 years	16 years	17 years	18 years	19 years	20 years	25 years
	*M* (*SD*)	*M* (*SD*)	*M* (*SD*)	*M* (*SD*)	*M* (*SD*)	*M* (*SD*)	*M* (*SD*)	*M* (*SD*)	*M* (*SD*)	*M* (*SD*)	*M* (*SD*)	*M* (*SD*)
Support												
O	3.16 (0.70)	3.25 (0.75)	3.24 (0.73)	3.31 (0.68)	3.42 (0.71)	3.88 (0.62)	3.36 (0.76)	3.35 (0.54)	3.40 (0.55)	3.33 (0.50)	3.40 (0.57)	3.86 (0.49)
U	3.08 (1.01)	3.00 (0.81)	3.15 (0.84)	3.38 (0.77)	3.40 (0.76)	3.86 (0.70)	3.12 (0.67)	3.19 (0.57)	3.31 (0.49)	3.39 (0.54)	3.28 (0.60)	3.89 (0.46)
R	3.45 (0.82)	3.51 (0.72)	3.49 (0.68)	3.63 (0.68)	3.59 (0.64	3.84 (0.81)	3.44 (0.68)	3.59 (0.49)	3.57 (0.51)	3.58 (0.44)	3.60 (0.60)	3.96 (0.52)
Neg. Int.												
O	1.26 (0.39)	1.37 (0.50)	1.29 (0.43)	1.26 (0.43)	1.25 (0.47)	1.58 (0.55)	1.33 (0.44)	1.32 (0.36)	1.25 (0.41)	1.27 (0.46)	1.21 (0.35)	1.53 (0.46)
U	1.31 (0.43)	1.55 (0.51)	1.49 (0.68)	1.41 (0.53)	1.42 (0.52)	1.50 (0.53)	1.28 (0.37)	1.33 (0.55)	1.21 (0.32)	1.18 (0.34)	1.21 (0.35)	1.55 (0.46)
R	1.15 (0.25)	1.24 (0.36)	1.28 (0.46)	1.20 (0.31)	1.19 (0.37)	1.45 (0.51)	1.26 (0.38)	1.23 (0.38)	1.13 (0.22)	1.07 (0.18)	1.14 (0.25)	1.49 (0.47)
Dominance												
O	1.77 (0.53)	1.89 (0.62)	1.82 (0.51)	1.84 (0.58)	1.78 (0.56)	2.01 (0.59)	1.74 (0.53)	1.76 (0.53)	1.81 (0.62)	1.72 (0.49)	1.73 (0.52)	1.95 (0.55)
U	1.70 (0.55)	1.98 (0.60)	2.09 (0.74)	1.87 (0.54)	1.81 (0.47)	2.03 (0.46)	1.61 (0.44)	1.72 (0.52)	1.78 (0.49)	1.84 (0.54)	1.77 (0.63)	2.04 (0.51)
R	1.58 (0.50)	1.67 (0.55)	1.65 (0.46)	1.62 (0.53)	1.64 (0.46)	1.88 (0.63)	1.68 (0.42)	1.74 (0.44)	1.66 (0.41)	1.57 (0.45)	1.60 (0.47)	1.89 (0.51)

*Note: M* (*SD*) = Mean (Standard Deviation). O = Overcontrollers. U = Undercontrollers. R = Resilients. Neg. Int. = Negative Interaction.

**Table 2:** Bivariate Intercorrelations between Relationship Quality Indicators.

		1	2	3	4	5	6	7	8	9	10	11	12	13	14	15	16	17	18
1	Support T1	-																	
2	Neg. Int. T1	−.18*	-																
3	Dominance T1	.12*	.18*	-															
4	Support T2	.54**	−.11*	.04	-														
5	Neg. Int. T2	−.12**	.38**	.13**	−.20**	-													
6	Dominance T2	.03	.15**	.38**	.14**	.30**	-												
7	Support T3	.44*	−.10*	.04**	.61**	−.13**	.06	-											
8	Neg. Int. T3	−.11*	.25**	.09*	−.12**	.27**	.18**	−.17**	-										
9	Dominance T3	−.04	.11*	.30**	.00	.17**	.53**	.04	.35**	-									
10	Support T4	.40**	−.01	.08	.46**	−.06	.03	.55**	−.09*	−.02	-								
11	Neg. Int. T4	−.18**	.20**	.07	−.21**	.24**	.11*	−.20**	.40**	.20**	−.17**	-							
12	Dominance T4	−.08	.07	.28**	−.07	.12**	.36**	−.04	.14**	.44**	.00	.33**	-						
13	Support T5	.34**	−.06	−.04	.37**	−.08	−.00	.45**	−.09	−.02	.51**	−.10*	−.02	-					
14	Neg. Int. T5	−.05	.19**	.16**	−.15**	.21**	.13**	−.13**	.30**	.24**	−.05	.45**	.25**	−.05	-				
15	Dominance T5	−.08	.07	.30**	−.08	.13**	.39**	−.07	.21**	.42**	−.09*	.20**	.51**	−.02	.37**	-			
16	Support T6	.10*	.04	−.01	.17**	.03	.06	.24**	.02	−.00	.26**	−.07	−.03	.29**	.02	.03	-		
17	Neg. Int. T6	.00	.11*	.08	−.01	.16**	.09*	.00	.14**	.09	−.09	.23**	.17**	.00	.18**	.15**	−.24**	-	
18	Dominance T6	−.12**	.05	.22**	−.07	.07	.25**	−.09	.09*	.22**	−.09*	.12**	.38**	−.04	.09*	.36**	.02	.36**	-

*Note:* Neg. Int. = Negative Interaction. *p<.05. **p<.01. T1–T5 referred to best friendship quality. T6 referred to romantic relationship quality.

**Table 3:** Standardized Parameter Estimates of the Structural Part of the Models Testing the Indirect Effect of Adolescent Personality Types on Young Adulthood Romantic Relationship Quality through Development of Friendship Quality throughout Adolescence.

	Support		Negative Interaction		Dominance	
Parameter^a^	YoungerCohortβ (*SE*)	OlderCohortβ (*SE*)	YoungerCohortβ (*SE*)	OlderCohortβ (*SE*)	YoungerCohortβ (*SE*)	OlderCohortβ (*SE*)
Effects of Personality on Friendship Quality						
O vs. R → I Friendship Quality	−.17*** (.05)	−.21*** (.06)	.14* (.06)	.12* (.06)	.22** (.07)	.22** (.06)
U vs. R → I Friendship Quality	−.22*** (.05)	−.30*** (.07)	.15* (.06)	.14* (.06)	.18* (.08)	−.06 (.09)
O vs. R → LS Friendship Quality	−.04 (.08)	−.04 (.08)	.01 (.06)	.02 (.10)	.01 (.09)	.01 (.09)
U vs. R → LS Friendship Quality	.12 (.08)	.13 (.08)	.04 (.06)	.06 (.10)	.15 (.11)	.34* (.12)
Effects of Personality on Romantic RelationQuality						
O vs. R → Romantic Relation Quality	.03 (.05)	.04 (.06)	.05 (.05)	.05 (.05)	.03 (.05)	.03 (.05)
U vs. R → Romantic Relation Quality	.03 (.04)	.04 (.06)	−.01 (.05)	−.01 (.05)	−.01 (.05)	−.01 (.05)
Effects of Friendship Quality on RomanticRelation Quality						
I Friendship Quality → RomanticRelation Quality	.41*** (.08)	.42*** (.08)	.30*** (.06)	.33*** (.08)	.38*** (.06)	.39*** (.06)
LS Friendship Quality → RomanticRelation Quality	.43* (.08)	.56*** (.10)	.32** (.08)	.23** (.06)	.35^†^ (.08)	.37^†^ (.08)
Indirect Effects						
O vs. R→ I Friendship Quality →Romantic Relation Quality	−.07** (.03)	−.09** (.03)	.04^†^ (.02)	.05^†^ (.03)	.09** (.03)	.08** (.03)
U vs. R→ I Friendship Quality →Romantic Relation Quality	−.09** (.03)	−.13** (.04)	.04* (.02)	.05* (.03)	.07*^b^ (.03)	−.02^c^ (.04)
O vs. R→ LS Friendship Quality →Romantic Relation Quality	−.02 (.04)	−.02 (.05)	.00 (.02)	.00 (.02)	.00 (.04)	.00 (.04)
U vs. R→ LS Friendship Quality →Romantic Relation Quality	.05 (.04)	.07 (.06)	.01 (.02)	.01 (.03)	.05^b^ (.06)	.12^c^ (.08)
Other parameters						
Gender → I Friendship Quality	.42*** (.04)	.55*** (.06)	−.23*** (.06)	−.23*** (.06)	−.08 (.06)	−.08 (.06)
Gender → LS Friendship Quality	−.16*** (.07)	−.17*** (.08)	.06 (.06)	.09 (.10)	−.05 (.08)	−.04 (.08)
Gender → Romantic RelationQuality	.06 (.05)	.08 (.06)	−.07 (.04)	−.08 (.05)	−.12* (.04)	−.12* (.04)
Correlation between I and LSFriendship Quality	−.62***^a^ (.06)	−.54** (.10)	−.48** (.07)	−.72** (.08)	−.27^a^ (.12)	−.12^b^ (.17)

*Note.* O vs. R = Overcontrollers compared to Resilients. U vs. R = Undercontrollers compared to Resilients. β (*SE*) = Standardized coefficient (*Standard error*). I = Intercept. LS = Linear Slope. ^†^ *p*<.10. *p<.05. **p<.01. ***p<.001. ^a^To avoid convergence problems, the variances of quadratic slopes were fixed at zero. Therefore, no regression parameters could be estimated in the structural part of the models. Superscripts ^b^and ^c^indicated that magnitudes of parameters were significantly different across the younger and the older cohorts, thus they were freely estimated across cohorts.

### Adolescent Personality Types and Adolescent Friendship Quality

Regarding our first research aim, the findings generally confirmed that the mean levels (i.e., intercepts) of adolescent friendship quality differed by adolescent personality types (Figures 2–4). In both cohorts, overcontrollers (unstandardized coefficients [Bs]* = *−0.22, *ps*<.001) and undercontrollers (Bs* = *−0.32, *ps*<.001) perceived significantly lower levels of support from their best friend during adolescence than resilients. Regarding negative interaction with best friend during adolescence, overcontrollers (Bs* = *0.08, *ps* = .04) and undercontrollers (Bs* = *0.09, *ps* = .02) had higher levels of negative interaction than resilients. Moreover, both overcontrollers (Bs* = *0.16, *ps*<.001) and undercontrollers (B* = *0.14, *p* = .03) perceived higher levels of dominance from their best friend during adolescence. For undercontrollers, however, this was found only for the younger age cohort.

Fewer effects were found of the personality types on the developmental changes (i.e., linear slopes). In fact, the only significant finding was that undercontrollers increased significantly faster in perceived dominance from their best friend than resilients (B* = *0.06, *p* = .01), in the older age cohort. These results were found while controlling for the fact that girls had significantly higher mean levels (Bs* = *0.54, *ps*<.001) and slower growth (Bs* = *−0.04, *ps* = .03) in perceived friendship support, and significantly lower mean levels of negative interaction with their best friend in adolescence (Bs* = *−0.13, *ps*<.001) than boys in both cohorts. Overall, the pattern was quite consistent. Whereas almost no differences were found in the developmental changes in friendship quality, resilients reported the highest-quality friendships over the course of adolescence, compared to their overcontrolled or uncontrolled age-mates.

### Indirect Effect of Adolescent Personality Types on Young Adults’ Romantic Relationship Quality, through Adolescent Friendship Quality

The second research aim was to determine whether personality types would be linked to romantic relationships through a developmental “spill-over” from earlier friendship quality to later romantic relationship. Indications were found for this indirect linkage, because each of the essential paths constituting this indirect effect was significant. That is, over and above the effects of personality on quality of adolescent friendship, we also found indications for transmission of friendship quality to later romantic relationship quality in emerging adulthood. In fact, these linkages were generally (moderately) strong. Path estimates (βs) from adolescent friendship quality to young adulthood romantic relationship quality ranged from .41 to .56 for support, from .23 to .33 for negative interaction, and from .35 to .39 for perceived dominance.

Moreover, the transmission of the quality of adolescent friendships to young adulthood romantic relationships was further supported when the indirect effect was tested using stringent bootstrapping methods. Despite the fact that there were no direct paths from adolescent personality types on perceived support from, negative interaction with, and perceived dominance from romantic partners in young adulthood (Bs ranged from −0.01 to 0.04, *ps>*.05), there were significant indirect effects of adolescent personality types on young adulthood romantic relationship quality through the mean levels of adolescent friendship quality. Specifically, resilients indirectly experienced higher mean levels of support from their romantic partners in emerging adulthood than overcontrollers (Bs* = *−0.10, *ps*<.001) and undercontrollers (Bs* = *−0.14, *ps*<.001), through mean levels of adolescent friendship support. This was found in both age cohorts. In terms of negative interaction, resilients across cohorts indirectly experienced less negative interaction with romantic partner in young adulthood than overcontrollers (Bs* = *0.04, *ps* = .08) and undercontrollers (Bs* = *0.05, *ps* = .04), through the mean levels of negative interaction with their best friend in adolescence. Moreover, overcontrollers in both age cohorts indirectly perceived more dominance from romantic partner during young adulthood than resilients (Bs* = *0.10, *ps* = .01), through the mean levels in perceived dominance from their best friend in adolescence. In addition, undercontrollers in the younger cohort indirectly experienced more dominance from their romantic partner during young adulthood than resilients (Bs* = *0.09, *ps* = .04), through the mean levels in perceived dominance from their best friend during adolescence.

Although these indirect linkages were not observed for the developmental changes in friendship quality, findings provide support for the idea of indirect effects of adolescent personality types on young adulthood romantic relationship quality, through friendship quality in adolescence. Together, these models explained 12% to 27% of variance in the quality of young adults’ romantic relationships. Explained variances were 14% and 26% for perceived support, 12% and 7% for negative interaction, and 23% and 27% for perceived dominance, for the younger and older cohorts respectively. This indicates medium to large effect sizes (*rs* ranged from .26 to .52) according to the standards set by Cohen [49].

## Discussion

The current study aimed to provide more insight into the associations between personality types (i.e., overcontrollers, undercontrollers, and resilients), friendship quality in adolescence, and romantic relationship quality in early adulthood. Results showed that both overcontrollers and undercontrollers had lower friendship quality during adolescence than resilients, as indicated by lower perceived support from, more negative interaction with, and more perceived dominance from their best friend. Further, adolescent personality types had an indirect linkage with romantic relationship quality during young adulthood, through perceived quality of friendship during adolescence. These findings suggest that individuals’ personality may play an important role in the continuity of quality of relationships with friends and romantic partners. Results indicate that individual differences in adolescent friendship quality could “spill over” to romantic relationship quality in young adulthood.

### Personality Types and Development of Friendship Quality in Adolescence

The current study indicates that adolescents with different personality types differed significantly in the mean levels of all friendship quality indicators across the whole period of adolescence. We also found that undercontrollers grew faster in perceived best friends’ dominance from middle to late adolescence. Perhaps, undercontrollers’ relatively disruptive and impulsive interpersonal behaviors [30] decrease their own opportunities to influence their friends during the transition to young adulthood, as such behaviors become less acceptable over the course of development [50]. As a consequence, they might gradually experience more dominance from their best friends than resilients or overcontrollers do. Except for this difference, we did not find differences in growth rates in any of the other friendship quality indicators by adolescent personality types. Together these findings therefore suggest that the differences in friendship quality by personality types lie mainly in the mean levels.

The results that overcontrollers and undercontrollers perceived lower support and more negative interaction from their best friend were in accordance with the findings in the prior studies covering shorter time spans in adolescence [10], [26]–[28]. Findings may add to the existing literature by showing that the distinct patterns of perceived support and negative interaction reported by the different personality types were rather persistent across the whole period of adolescence. The reasons behind the relatively higher relationship quality for resilients are not yet clear. One prior study reported that resilients had better understanding of the conceptions of friendship, such as the meaning of closeness and trust between friends, conflict resolution among friends, and the processes through which people become friends [51]. It might be that resilients’ more mature understanding of friendship increases their capabilities for developing and maintaining friendships, and for experiencing more satisfactory friendships, compared to overcontrollers and undercontrollers.

Overcontrollers perceived more dominance from their best friend than resilients throughout adolescence. That is, overcontrollers were less likely to be the leader and take charge in their friendships than resilients. This finding is in accordance with the findings that overcontrollers are generally more compliant during conflicts with their best friend and experience more coercion from their friend than resilients [10], [28]. The findings are also line with a study reporting that overcontrollers were particularly vulnerable to their best friend’s influence in delinquent behaviors [29]. There might be two reasons for the overcontrollers’ “follower” position in their friendship. First, it might be due to overcontrollers’ low social potency: They are found to be more submissive, not fond of leadership roles, and to have little desire to influence others [9]. As such, it is possible that overcontrollers do not mind if their interpersonal partner (e.g., friend) takes charge in the relationship and dominates them. The other reason could be their low decision-making ability. A recent study has found that overcontrollers scored relatively high on indecisiveness [52], and it could well be that their friends therefore need to take charge and make decisions for them in the relationship. These two reasons might explain the finding that overcontrollers are more likely to be dominated in their friendships and follow their friends’ delinquent behaviors.

Similar to overcontrollers, undercontrollers also perceived more dominance from their best friend than resilients from early to middle adolescence. This result is in contrast to prior findings indicating that undercontrollers were not different from resilients in their reports of being forceful and being fond of leadership roles, and that they seemed to be capable of influencing delinquent behaviors of their best friend [29], [53]. The finding, however, is in agreement with studies reporting that undercontrollers were more compliant during conflict with their best friend and perceived more coercion in their friendship than resilients [10], [28]. It is intriguing that these two lines of evidence regarding undercontrollers’ influence on their friend are contradictory. One possible interpretation of these results is that although undercontrollers generally have the propensity of assuming leadership roles and have the desire to influence others, they are only capable of influencing their best friend with actual behaviors such as delinquency. They do not seem to be proficient in impacting their friend by presenting convincing arguments. One possible reason might be that undercontrollers lack social skills [53], and therefore are not able to persuade their friend to follow their suggestions. Another reason could be that, like overcontrollers, their relatively higher level of indecisiveness compared to resilients [52] puts their friend in the position of making decisions in the relationship. In sum, undercontrollers might unintentionally impact their friends’ behavior, as their risk-taking may be regarded as evidence of independence and maturation during adolescence, and thus appear attractive to and be copied by their friends [54]. However, undercontrollers might not be able to purposefully influence their friends.

### Indirect Effect of Personality Types on Romantic Relationship Quality through Friendship Quality

Our results suggest that adolescent personality types could potentially have an impact on romantic relationship quality during young adulthood, although this impact appears to be indirect, through friendship quality during adolescence. Consistent with prior studies, our study showed that overcontrolled children did not directly differ from resilients in romantic relationship quality during young adulthood [9], [30]. In contrast, we did not find that undercontrollers directly had more conflicting romantic relationships during young adulthood than resilients.

Although we did not find direct effect of personality types on romantic relationship quality, indirectly, however, undercontrollers, as well as overcontrollers, experienced lower romantic relationship quality than resilients. Specifically, overcontrollers’ and undercontrollers’ lower friendship quality during adolescence, as compared to resilients’, was subsequently related to lower romantic relationship quality during young adulthood. Our study suggests that individuals’ differential levels of quality in friendship during adolescence tend to “spill over” to different levels of quality in romantic relationship during young adulthood. These “spill-over” effects were further suggested by the consistently moderate, significant linkages between friendship quality and romantic relationship quality five years later. These findings provide more insight into potential mechanisms underlying how personality may affect romantic relationship quality. Perceived friendship quality during adolescence might be one of the underlying processes linking personality and perceived romantic relationship quality during emerging adulthood.

Two explanations could be provided for this potential mechanism. First, from an attachment perspective, youths may develop expectancies for interpersonal relationships based on their earlier close relationships [33], [55]. These expectancies form mental representations (working models) of the self and relationship partners that guide interaction patterns in their later relationships, including romantic ones [56]–[58]. Resilients perceived relatively higher friendship quality in adolescence, and might thus develop representations of themselves as desirable and skillful interpersonal partners. However, overcontrollers and undercontrollers who had lower friendship quality in adolescence might develop internal representations of themselves as undesirable interpersonal partners. These differential expectations, based on earlier interpersonal relationships, might affect their romantic relationship quality. Second, friendships might serve as a place where youths can practice social skills in egalitarian and reciprocal relationships [6], [32]. These findings thus suggest that overcontrollers and undercontrollers may not have the opportunities to develop such skills in the friendship context during their adolescent years. Therefore, overcontrollers and undercontrollers might enter romantic relationships with fewer social skills learned from prior friendships than resilients, such as abilities to establish intimacy, negotiate in conflict, and balance dominance with their best friend. Ultimately, due to their own poorer relationships skills, they could also end up in romantic relationships of poorer quality. These two potential explanations could unfortunately not be tested in the current study, but are an important area for future research.

### Strengths, Limitations, and Future Research Directions

This study has several strengths. It followed two age cohorts of youths over a period of ten years. This allowed us for the first time to examine the link between personality and development of friendship quality throughout adolescence. In addition, the current study provides more insight into the mechanism underlying the link between personality and romantic relationship quality. That is, by integrating personality, friendships, and romantic relationships into one model, this study revealed that personality predicted later romantic relationship through earlier friendship quality development.

Despite these strengths, some limitations of the current study should be mentioned. One limitation lies in the use of single-informant data, which might introduce reporter bias.

Both friendships and romances are dyadic relationships, within which each person’s perceptions and behaviors are important factors to consider. Prior research has shown that the degree of similarity between friends’ and partners’ personality influences the quality of their relationship [59]. Thus future research could explore the relationships between the variables using data from various sources (e.g., both participant and their best friend and romantic partner) to capture a more complete picture. Second, even though we found longitudinal associations between adolescent personality, adolescent friendship quality, and young adulthood romantic relationship quality, we cannot draw causal conclusions due to the design of the study.

## Conclusion

Taking together, the current study extends previous research by showing that individuals with different personality types differed in their mean levels of friendship quality during the whole period of adolescence, and through these differences, they might indirectly experience different levels of romantic relationship quality during young adulthood. These findings illustrate the complex processes by which personality might affect quality of close social relationships in the short run, and the longer run. They suggest a developmental sequence in which individuals’ personality predicts proximal friendship quality during adolescence, and this in turn predicts distal romantic relationship quality during young adulthood.
